# Distal Flow Diversion with Anti-Thrombotically Coated and Bare Metal Low-Profile Flow Diverters—A Comparison

**DOI:** 10.3390/jcm12072700

**Published:** 2023-04-04

**Authors:** Marie-Sophie Schüngel, Karl-Titus Hoffmann, Erik Weber, Jens Maybaum, Nikolaos Bailis, Maximilian Scheer, Ulf Nestler, Stefan Schob

**Affiliations:** 1 Abteilung für Neuroradiologie, Klinik & Poliklinik für Radiologie, Universitätsklinikum Halle, 06120 Halle (Saale), Germany; marie-sophie.schuengel@uk-halle.de; 2 Institut für Neuroradiologie, Universitätsklinikum Leipzig, 04103 Leipzig, Germany; 3 Klinik für Anästhesie und Notfallmedizin, Universitätsklinikum Leipzig, 04103 Leipzig, Germany; 4 Abteilung für Neurochirurgie, Universitätsklinikum Halle, 06120 Halle (Saale), Germany; 5 Klinik und Poliklinik für Neurochirurgie, Universitätsklinikum Leipzig, 04103 Leipzig, Germany

**Keywords:** flow diversion, low-profile flow diverter, small cerebral vessels, Silk Vista Baby, p48MW, coating

## Abstract

Background and purpose: The establishment of low-profile flow diverting stents (FDS), for example, the Silk Vista Baby (SVB) and the p48MW, facilitated endovascular treatment of peripheral cerebral aneurysms. This study therefore aims to compare the performance and outcomes of the SVB with those of the p48MW HPC, with a special focus on hemodynamic aspects of peripheral segments and bifurcations. Materials and methods: The study cohort comprises 108 patients, who were either treated with the SVB or the p48MW HPC between June 2018 and April 2021. Results: Sixty patients received a SVB and forty-eight patients a p48MW HPC. The SVB was used predominantly in the AcomA-complex, and the p48MW HPC in the MCA bifurcation. Immediately after implantation, significant hemodynamic downgrading (OKM A2-A3, B1-B3, C3) was achieved in 60% in the SVB group vs. 75.1% in the p48MW HPC group. At the second follow-up, after an average of 8.8 and 10.9 months, respectively, OKM D1 was observed in 64.4% of the SVB group vs. 27.3% in the p48MW HPC group. Only 1.7% vs. 6.8% of the aneurysms remained morphologically unaltered (OKM A1). Adverse events with persisting neurologic sequalae at last follow-up were largely comparable in both groups (5.0% vs. 4.2%). Conclusion: Immediately after implantation, the p48MW HPC had a more profound hemodynamic impact than the SVB; however, early complete occlusions were achieved in a greater proportion of lesions after implantation of the uncoated SVB.

## 1. Introduction

Flow diversion (FD) has emerged as a reliable, minimally invasive therapeutic concept for cerebral aneurysms [1]. The first FDS were designed and approved exclusively for proximal intracranial aneurysms of the anterior circulation, i.e., the intracranial internal carotid artery from the petrous to the clinoid segment [2]. However, accompanying the success of this approach, challenging indications—for example, post-bifurcational segments of the distal anterior circulation and vertebrobasilar aneurysms—are increasingly considered for flow diversion [3,4,5,6].

FDS suitable for the treatment of distal segments of the Circle of Willis require a set of distinct features compared to conventional FDS. For example, catheterization of those segments necessitates well maneuverable, small microcatheters, and, hence, the profile of the corresponding FDS must be comparatively low in order to remain implantable via the latter. Addressing this, low-profile FDS, for instance, the Silk Vista Baby (SVB; Balt, Montmorency, France) and the p48MW (phenox, Bochum, Germany), were developed and are now available for on-label use in small cerebral vessels [7,8]. Both devices are designed for vessel segments ranging from 1.5 mm to 3 mm in diameter and are composed of 48 drawn filled tubing (DFT) strands with an outer nitinol shell and an inner platinum core, the latter providing the required radiopacity for implantation. The SVB is compatible with a 0.017” microcatheter, whereas the p48MW requires a 0.021” microcatheter for delivery, but comes with the feature of an independently movable wire (MW), which can be placed 6 cm distal to the target segment for stabilization during implantation in difficult anatomical circumstances [9].

The high surface area coverage of FDS, accounting for approximately 30% of the parent vessel surface, is associated with significant thrombogenicity in vivo [10,11]. Therefore, implantation of FDS requires sufficient inhibition of platelet function in order to avoid thromboembolic events, which is conventionally achieved by administration of dual anti-platelet therapy (DAPT) [12]. The latter, however, may cause critical hemorrhagic complications [13]. A promising approach to the problem of balancing the risk of thromboembolic complications against the risk for hemorrhagic complications associated with FD is the application of surface coating technologies on FDS. One of these biomimicry technologies, for example, uses phosphorycholine, a component of the red blood cell membrane, to cover the surface of the FDS in order to improve its hemocompatibility [14]. In that way, platelets do not adhere to the foreign material of the FDS and the physiological coagulation homeostasis is maintained [10].

Of the currently approved low profile FDS, the p48MW is the only one that is available with an anti-thrombotic hydrophilic polymer coating ‘HPC’, which distinctly reduces adhesion of platelets [15]. As a consequence, the HPC version of the p48MW allows earlier reduction of DAPT, or even the application of single anti-platelet therapy (SAPT) if the clinical situation demands it [16,17].

Aside from the surface modification of the p48MW HPC the architectures of the SVB and the p48MW are largely comparable. Nevertheless, recent reports indicate that each of the devices may have specific suitability profiles for different cerebrovascular segments, and that time span to aneurysm occlusion post implantation may differ between both devices [8,18]. Therefore, the aim of this study is (a) to report our experiences with both devices for treatment of distally located cerebral aneurysms specifically focusing on their eligibility for different locations and (b) to provide a first comparison of the efficacy, clinical outcomes and angiographic outcomes after implantation of the SVB and the p48MW HPC.

## 2. Materials and Methods

### 2.1. Ethics Approval

The retrospective analysis of a prospectively maintained database including patients between June 2018 and April 2021 was approved by the institutional ethics committee (local IRB no AZ 208-15-01062015). Informed consent of each patient regarding the scientific use of radiological and clinical data was obtained in writing either from the patient himself/herself or his/her legal representative.

### 2.2. Study Design

The prospectively curated institutional database, which includes all endovascular treatments between November 2013 and March 2021, was reviewed to identify all procedures performed employing the Silk Vista Baby or the p48MW HPC, or a combination of both devices. Aneurysms with saccular, blister-like and fusiform morphology were included. Patients with acutely ruptured dissecting aneurysms were not included in this study.

Demographic data, localization, size and morphology of each target lesion, technical and clinical adverse events as well as angiographic follow-up data were collected. Table 1 provides an overview of our patient database.

### 2.3. Anti-Platelet Regimen

DAPT was initiated in all but three patients. The loading doses of 500 mg acetylic salicylic acid (ASA) and either 180 mg ticagrelor (Brilique, AstraZeneca, Hamburg, Germany) or 30 mg prasugrel (Efient, Orifarm, Leverkusen, Germany) or 300 mg clopidogrel (Plavix, 1A Pharma, Holzkirchen, Germany) were administered 24 h prior to the intervention. In emergency cases, however, patients received a bolus of 500 mg ASA intravenously (i.v.) at the beginning of the intervention. In addition, a bolus of body-weight adapted Eptifibatide i.v. (Integrilin, 180 µg/kg; GlaxoSmithKline, Ireland) was given to bridge the duration of the intervention before the second anti-platelet agent was amended orally, immediately after the intervention.

DAPT was then continued as a combination of 100 mg ASA with either ticagrelor 180 mg (given in two single doses of 90 mg 12 h apart) or 10 mg prasugrel or 75 mg clopidogrel daily for at least 12 months, followed by a life-long monotherapy with ASA.

In three cases, a decision was made to keep the patients on SAPT only. The rationale for SAPT in these cases, which demanded a less aggressive inhibition of thrombocyte function, was a preexisting anticoagulation due to cardiologic indication in two patients who remained on ASA only. In the third case, patient anti-platelet therapy with prasugrel only was administered with regards to imminent renal transplantation.

### 2.4. Endovascular Treatment

All procedures were performed under general anesthesia using a biplane angiography system (Philips AlluraClarity, Best, The Netherlands). An eight French introducer sheath (Terumo radifocus II, Leuven, Belgium) was established via the right common femoral artery and a bolus of 5000 international units of heparin (ratiopharm, Ulm, Germany) was given via the sheath. For triaxial access the guiding catheter was introduced to the respective supra-aortic target vessel with the use of a 5F diagnostic catheter (Cordis TEMPO AQUA) in either Vertebral or Simmons 2 configuration. Either the Neuron Max 088 (6F; Penumbra, Alameda, CA, USA) or the Cerebase (Cerenovus, Irvine, CA, USA) were used as guiding catheters. A 6F Sofia in 115 cm was used as distal access catheter (MicroVention Terumo, Aliso Viejo, CA, USA) in order to increase support for device delivery.

#### 2.4.1. Microcatheters Used for Delivery of the Silk Vista Baby

Depending on the localization of the aneurysm, vessel anatomy and size, different microcatheters were used for implantation of the SVB. In cases of a proximally located aneurysm—i.e., the internal carotid artery (ICA) terminus, the M1 segment of the middle cerebral artery (MCA) or the vertebral artery—the Headway 17 (0.017′; MicroVention Terumo, Aliso Viejo, CA, USA) or the Gama 17 (0.017′; Balt, Montmorency, France) were used. The Headway 17 was initially recommended as delivery catheter; the Gama was specifically designed for the SVB but was released only recently.

In case the target lesion originated from a small peripheral vessel, for example, the pericallosal or callosomarginal artery, or from a challengingly configured complex of the anterior cerebral and anterior communicating arteries, the Excelsior SL10 (0.0165′; Stryker Neurovascular, Cork, Ireland) was used. The latter, however, was only suitable for implantation of the small variants of the SVB with a diameter of 2.25 mm and 2.75 mm, respectively, as described in a prior report [7].

#### 2.4.2. Microcatheters Used for Delivery of the p48MW HPC

The p48MW HPC requires a 0.021′ microcatheter for delivery. In the majority of patients, the Prowler Select Plus (0.021′; Cerenovus, Irvine, CA, USA) was used. However, related to repeated difficulties during catheterization of challengingly curved vessels, the Headway 21 (0.021′; MicroVention Terumo, Aliso Viejo, CA, USA) was tried and then used as a favored microcatheter for p48MW delivery in later interventions.

### 2.5. Post-Interventional Course and Follow-Up

After the intervention, all patients were transferred to the intensive care unit (ICU) ensuring continuous monitoring for a minimum of 24 h and neurological examination. Non-enhanced cranial computed tomography (CCT) was performed within 24 h post-interventionally in every case.

Routinely, radiography was then performed 4 weeks after FDS implantation for assessment of possible device-induced vasospasm [19]. Angiographic follow-ups were planned at 3, 9 and 24 months after the intervention [20].

## 3. Results

### 3.1. Baseline Characteristics

A total of 108 patients (33 men and 75 women; average age of 56.3 years) harboring 114 lesions met the inclusion criteria and were comprised in our analysis. Therefrom, the majority of aneurysms (110/114; 96.5%) were of the saccular side-wall type, 1.8% were blister-like aneurysms and 0.9% were fusiform aneurysms.

The target lesions were distributed as follows: aneurysms were most commonly located in the anterior circulation (100/114; 87.7%). Of them, 21% (21/100) originated from the terminus of the internal carotid artery including the posterior communicating artery (PcomA), 44% (44/100) from the anterior cerebral artery (ACA) starting with the A1-A2 junction also including distal segments, such as the pericallosal artery, and 35% (35/100) from the middle cerebral artery. The remaining lesions (14/114; 12.3%) were located in the posterior circulation. Of those, 35.7% (5/14) arose from the vertebral artery including the PICA orifice, 57.1% (8/14) from the basilar artery, and one aneurysm (7.1%) was located at the posterior cerebral artery.

On average, 1.12 FDS per patient were implanted. In 93 patients a single flow diverter was sufficiently implanted; 11 patients required deployment of two FDS to ensure sufficient treatment of the target lesion. In four cases, however, three or more devices were implanted in telescoping technique.

### 3.2. Comparison of the SVB and p48MW HPC: Patients and Treatments

More than half of the patients (*n* = 60) were treated with the SVB, the remaining 48 patients were treated with the p48MW HPC. The following differences regarding demographic data and distribution of the aneurysms were observed between the groups: on average, the patients treated with the SVB were younger (54.1 years vs. 58.5 years for the p48MW HPC group) and were more frequently male. The majority of aneurysms in the SVB group originated from the anterior cerebral artery including the pericallosal artery (56.9%). The p48MW HPC group had more than half of the aneurysms in the MCA complex (55.1%). Endovascular treatments in the posterior circulation were more frequently performed using the p48MW HPC. More specifically, 16.3% of the treatments in the p48MW HPC group were performed in the posterior circulation, whereas only 9.2% in the SVB group had posterior circulation aneurysms. Table 2 provides an overview of the anatomical distribution of the respective aneurysms.

The lesion morphology also differed between the two groups. All patients who were treated with the SVB suffered from saccular shaped side-wall aneurysms. In the p48MW HPC group, in contrast, a slightly smaller proportion (91.8%) of the patients were suffering from saccular side-wall aneurysms. A total of 4.1% had blister-like aneurysms and, respectively, 2.1% had fusiform aneurysms.

Flow diversion was successful in all cases. However, supplementary devices were required in 8.3% (5/60 in the SVB group) vs. 4.2% (2/48 in the p48MW HPC group) of the patients to ensure sufficient treatment of the target lesion. In a total of three patients, who presented with exceptionally large-sized aneurysms, additional coils were loosely deployed within the aneurysm dome prior to FDS implantation aiming to facilitate thrombus formation.

In two cases implantation of further FDS was required. These were related to a patient suffering from a large, broad-based aneurysm arising from the right-sided C6 segment of the internal carotid artery, consequently included in both treatment groups. Due to distal retraction of the initially implanted SILK + (Balt, Montmorency, France) and distinctly varying diameters of the vessel segments proximal and distal to the target lesion, supplementary FDS including the SVB as well as the p48MW HPC were implanted in plug and pipe technique to sufficiently cover the aneurysm neck. In another patient presenting with a wide-necked aneurysm arising from the anterior communicating artery, flow-T stenting was considered as the most promising treatment approach [21]. Therefore, the bilateral distal A2 segments were probated. Firstly, the SVB was deployed into the contralateral A2 segment, proximally ending at the level of the aneurysm neck. Coils were implanted in the bifurcation aneurysm. The LEO+ Baby stent (Balt, Montmorency, France) was then implanted into the ipsilateral A1–A2 junction. One patient presented with a saccular aneurysm of the basilar artery (BA) tip and an additional endoluminal stenosis of the BA segment proximal to the aneurysm with hemodynamic impact to the downstream territory. Therefore, a balloon-expandable coronary stent (REBEL, Boston Scientific) was implanted beyond the SVB to achieve a functionally satisfying result.

### 3.3. Adverse Events

#### 3.3.1. Technical Challenges

In the SVB group, retraction or distinct foreshortening of the implanted device immediately after deployment occurred in five patients (8.3%). In all these cases, implantation of a second SVB in telescoping technique was required. Device shortenings were either the result of undersizing the implant or of significant elongation and the tortuous course of the target vessel. However, none of the patients suffered from clinical sequelae related to the initial technical obstacles. In one further patient suffering from an incidental aneurysm arising from the origin of the posterior communicating artery, the SVB was successfully implanted into the ipsilateral ICA. However, distal device shortening was detected at the second angiographic follow-up five months post-intervention resulting in insufficient coverage of the aneurysm neck. Re-treatment was necessary and then performed using a second-generation Pipeline Embolization Device (PED2; Medtronic, Covidien, Minneapolis, MN, USA).

In patients treated with the p48MW HPC, technical adverse events were observed in seven cases (14.6%). In two, the FDS showed insufficient opening with incomplete wall apposition immediately after deployment. Additional angioplasty using a compliant balloon (Scepter C; MicroVention Terumo, Aliso Viejo, CA, USA) was performed and resolved the issue in both cases. Dislocation of the p48MW HPC during implantation occurred in three patients. Therefrom, in two patients, the FDS was completely recaptured, removed and substituted by another p48MW HPC. In the third patient, the p48MW HPC dislocated immediately after deployment and required implantation of two further FDS in plug and pipe technique to ensure adequate coverage of the aneurysm neck. Deployment of the second device was uneventful in each case. In the two remaining patients, the FDS had markedly shortened immediately after implantation as a consequence of tortuous segmental anatomy. However, there were no clinical manifestations associated with these technical events.

In one patient who suffered from a fusiform aneurysm of the basilar artery tip, one p48MW was implanted using the proximal P1 segment of the right posterior cerebral artery as distal landing zone and the distal third of the basilar artery as proximal landing zone. Although the patient did not suffer from any neurological deficits at any time, the patient’s first angiographic follow-up five months after treatment revealed a significant stenosis of the treated segment at the PCA orifice. In order to prevent ischemic complications, balloon angioplasty was performed, but did not result in significant improvement of the lesion. Therefore, a coronary stent (Rebel; Boston Scientific, Maple Grove, MN, USA) was implanted and achieved permanent reconstruction of the vessel. The patient remained clinically asymptomatic.

#### 3.3.2. Peri-Interventional Adverse Events

In the SVB group four adverse events (6.7%) occurred; three were ischemic and one was hemorrhagic. Ischemic events included transient side branch occlusion and distinctly delayed perfusion of the territory distal to the FDS as well as thrombus formation between two implanted FDS. In all cases, an intravenous bolus of body-weight adapted Eptifibatide (GlaxoSmithKline, Ireland) was administered immediately, followed by continuous infusion for 24 h. In the case of thrombus formation between the not completely adapted layers of two telescopically implanted flow diverter stents, additional angioplasty (Scepter C) was performed and resulted in improved alignment of the FDS with each other and with the vessel wall. In another patient exhibiting two incidental aneurysms of the paraophthalmic ICA, the injection after microcatheter probation revealed contrast extravasation from the aneurysm sack. The SVB was rapidly deployed sufficiently covering the target segment. In the control injection, the extravasation had stopped. Minor subarachnoid hemorrhage (SAH) was revealed by the control cCT the day after the intervention; however, the patient did not suffer from any clinical sequelae.

In the p48MW HPC group, a distinct delay in perfusion of the covered side branch immediately after FDS implantation was observed in one case (2.1%). In order to prevent any ischemic events, a bolus of Eptifibatide was administered as described above. Perfusion normalized completely and the patient did not show any neurological sequelae post intervention.

#### 3.3.3. Clinical Adverse Events

The SVB group

Adverse events in the SVB group causing clinically relevant sequelae occurred in eight of sixty patients (13.3%). However, the majority of patients recovered completely. At the last clinical follow-up, only three patients (5.0%) still presented with neurological impairment. Additionally, no treatment-related deaths within were observed during the study period.

In two of the eight patients, transient cerebral ischemia manifested clinically after stent occlusion in the early post-interventional course, within 72 h after the treatment. Both received a bolus of body-weight adapted Eptifibatide i.v. In one patient, the thrombus resolved completely together with the patient’s neurologic symptoms comprising an acute partial hemiparesis. In the second patient, the SVB remained occluded despite rescue therapy with intravenous Eptifibatide; however, the network of leptomeningeal collaterals connecting the ACA- and MCA periphery maintained perfusion of the affected territory in a retrograde fashion. At the last clinical follow-up, the patient showed good recovery and presented only subtle residual speech disturbances (mRS1).

Subacute device-induced vasospasm with temporary clinical manifestations, a phenomenon reported only recently [19], manifested in two patients without permanent neurological deficits.

Ischemic infarction in the early post-interventional course after FDS implantation was observed in two individuals. The first of these patients had mild aphasia after endovascular treatment of an aneurysm arising from the left-hand side pericallosal artery. Control cCT revealed areas of infarction in the unilateral territory of the ACA and a small part of the anterior third of the MCA territory. However, the neurologic deficits resolved completely within few days, prior to discharge from the hospital. In the other patient, revision treatment of a relapsed AcomA-aneurysm after coiling was performed by implanting the FDS in crossover-technique from the left-hand side A1 segment into the right-hand side A2 segment. Despite technical successful deployment, the control injection 15 min after implantation revealed a delayed perfusion of the covered left-sided A2 segment. Eptifibatide was administered immediately, significantly improving the perfusion of the downstream ACA territory. Still, the patient presented with severe hypodynamic delirium six hours after the intervention. Subsequently initiated cCT revealed partial bilateral infarctions of the ACA- and MCA territories, most likely related to peri-interventional microembolism in the course of the extended procedure time. Despite that, the patient showed good recovery (mRS1) until last clinical follow-up and only presented residual transient speech disturbance.

One case of acute frontobasal parenchymal hemorrhage causing subfalcine herniation occurred 12 h after FDS implantation demanding immediate craniotomy. Despite this major complication, the patient showed an almost complete recovery a few months later, only presenting with mild residual difficulties in walking at the last clinical follow-up (mRS 2). Another patient exhibiting an unstable, partially thrombosed aneurysm of the left-sided MCA experienced a brachial hemiparesis as a result of increased perifocal edema of the aneurysm after implantation of the SVB. Prophylactic corticosteroids had already been started prior to the intervention, which was then amended by Celecoxib 100 mg (Celebrex, Kohlpharma GmbH, Merzig, Germany) daily. The paresis resolved completely within five days.

The p48MW group

In the p48MW HPC group, clinical impairment post intervention was observed in eight patients (16.6%); however, at the last clinical follow-up, only two (4.2%) still presented residual neurological deficits. Comparable to the SVB, treatment-related mortality did not occur within this group.

Three of these eight patients had hemorrhagic complications. Intracranial hemorrhage in the aftermath of FDS implantation occurred in two cases. One patient who underwent revision treatment after flow diversion of a left vertebral artery (V3-V4) dissecting aneurysm presented with prolonged postoperative recovery together with newly developed anisocoria after extubation despite a technically uneventful intervention. The immediate cCT revealed a remote cerebellar hemorrhage which was surgically evacuated. The other patient, who was treated for a MCA-bifurcation aneurysm, suffered from right-frontal intracerebral hemorrhage within 72 h after endovascular therapy, demanding an external ventricular drainage in the early phase. The third patient developed epistaxis and hematemesis in the early post-interventional course most likely related to a Mallory–Weiss lesion that was exacerbating under DAPT. However, two out of three patients recovered completely from the hemorrhage. Only one kept mild neurologic deficits (mRS1) up to the last available clinical follow-up.

In one patient cerebral ischemia manifested after successful treatment of a MCA bifurcation aneurysm within 24 h after the intervention. Although a body-weight adapted bolus of Eptifibatide was administered immediately, the matters were further complicated by the patient’s need for an oral anticoagulation due to a cardiologic indication. After secondary hemorrhagic transformation of the partial MCA infarction, protective hemicraniectomy was required. At the last clinical follow-up, the patient still presented moderate neurological impairment comprising hemiparesis and speech disturbance (mRS3).

Device-induced subacute vasospasm without permanent sequelae manifested in one patient. Two patients experienced minor stroke within four months after implantation of the p48MW HPC manifesting with a transient hemiparesis. MRI revealed subtle embolic infarcts in both patients. Both patients recovered completely during the initial hospital stay. In a patient suffering from a giant basilar artery aneurysm, inflammatory changes of the aneurysm wall with MRI contrast enhancement and perifocal edema caused brain stem affection 18 months after the procedure. Neurological impairment resolved completely after anti-inflammatory therapy with cortisone.

### 3.4. Angiographic Outcome

#### 3.4.1. Hemodynamic Changes Immediately after Flow Diversion

In the SVB group, the majority of the treated lesions (47.7%) immediately showed a marked delay in aneurysm perfusion (OKM A2-A3). Moreover, in 10.8% the aneurysm dome remained only partially perfused corresponding to OKM B1-B3. In one case (1.5%) of a peripheral MCA aneurysm arising from a small side branch of the superior trunk, a very profound hemodynamic effect was observed with a neck remnant only (OKM C3). However, 40% of the aneurysms did not show any immediate hemodynamic changes (OKM A1).

In the p48MW HPC group, the great majority of the treated lesions (68.8%) showed a prolonged stasis of contrast agent within the aneurysm dome (OKM A2-A3) immediately after implantation of the flow diverting stent. In 6.3% of the cases, the target lesion remained only partially perfused (OKM B1-B3). No observable changes in hemodynamics (OKM A1) were seen in 25% of the treated lesions.

#### 3.4.2. Hemodynamic Changes after the First Angiographic Follow-Up

The first angiographic follow-up after an average of 3.1 months and 3.9 months, respectively, was available in 54 patients (59 aneurysms) of the SVB group and in 43 patients (44 aneurysms) after treatment with the p48MW HPC.

More than half of the aneurysms (52.5%) treated with the SVB were already completely occluded, according to OKM D1. A further 33.9% revealed a distinct decrease in perfusion of the aneurysm dome (OKM B-C), and 8.5% showed delayed perfusion (OKM A2-A3). Only 5.1% of the treated lesions remained morphologically unaltered according to OKM A1.

**Figure 1:**
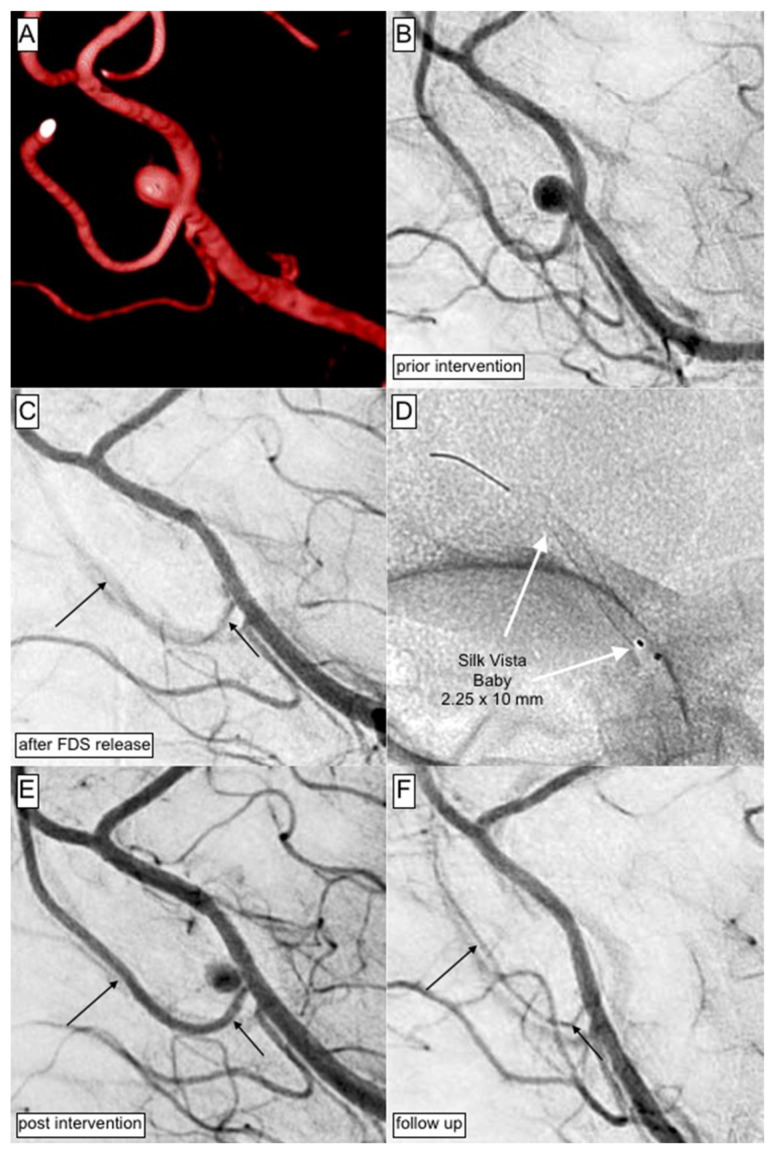
Shows the endovascular therapy of an incidental aneurysm in the distal ACA with the Silk Vista Baby flow diverter. (**A**) The reconstructed 3D angiogram demonstrates a saccular, broad-based aneurysm arising from the left-handed distal ACA at the level of the pericallosal artery. (**B**) Conventional DSA in working projection prior to the FDS implantation. (**C**) The SVB was implanted without any technical obstacles; however, diminished opacification of the covered callosomarginal artery was observed immediately afterwards and required rescue treatment with a GPIIb/IIIa inhibitor. (**D**) Correct position and complete wall adaptation of the implanted device sufficiently covering the aneurysm neck. (**E**) After administration of a body-weight adapted bolus of Eptifibatide intravenously, the control angiogram showed restored perfusion of the covered branch and the downstream territory. In the aftermath of the intervention, the patient did not suffer from neurological deficits. (**F**) At the first angiographic follow-up three months after FDS implantation, the covered segment shows distinct narrowing and the target aneurysm is completely occluded (OKM D1).

An exemplary case of successful aneurysm treatment using the SVB low-profile flow diverter is shown in Figure 1.

Compared to these results, a significantly smaller proportion of aneurysms treated with the p48MW HPC was completely occluded at first follow-up (OKM D1; 22.7%). The vast majority of lesions (52.3%) revealed decline in perfusion and aneurysm size according to OKM B-C.

#### 3.4.3. Hemodynamic Changes at the Second Angiographic Follow-Up

The second follow-up was performed after a mean of 8.8 months and 10.9 months, respectively. The rates of complete aneurysm occlusion (OKM D1) significantly increased in both treatment groups (64.4% vs. 27.3% in the p48MW HPC group). Only 1.7% of the aneurysms after SVB implantation and 6.8% after p48MW HPC treatment still remained morphologically unaltered.

Detailed information concerning the timeline of aneurysm occlusion in the individual cases is given in Table 2.

**Figure 2:**
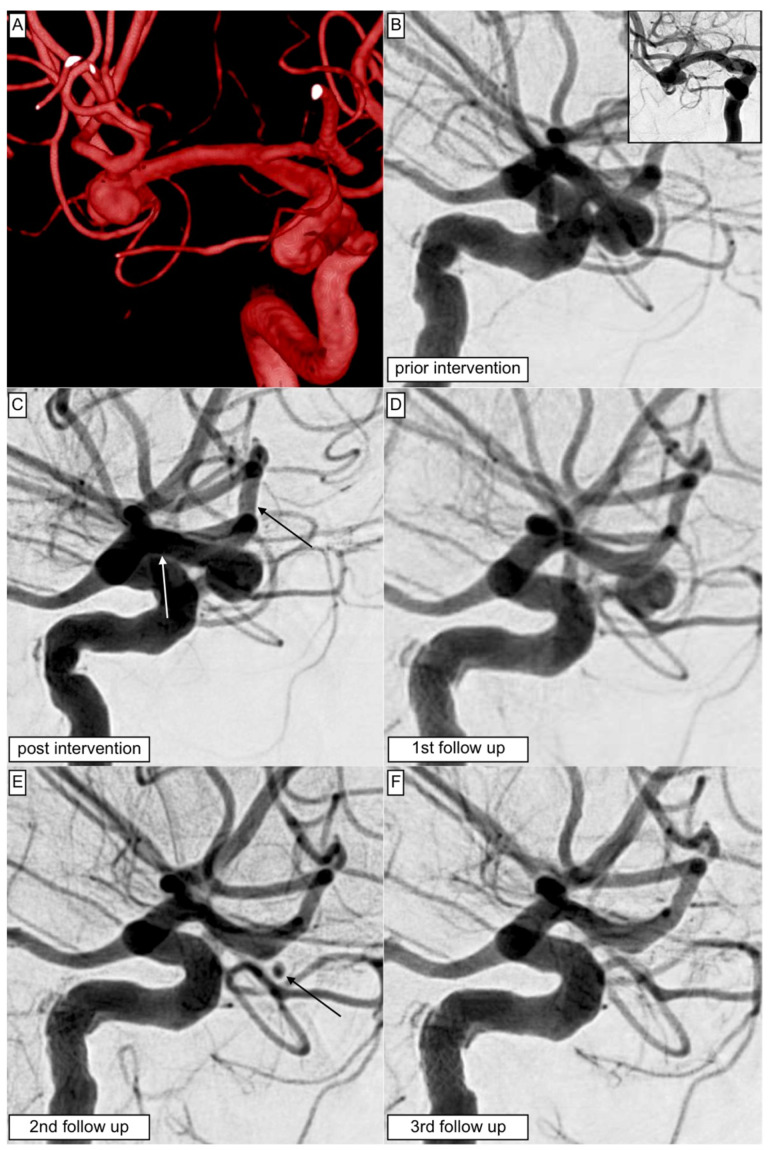
Shows the primary treatment of an irregular-shaped aneurysm arising from the right-hand side MCA trifurcation using the p48MW HPC: (**A**) The reconstructed 3D angiogram demonstrates the broad neck of the aneurysm completely comprising the MCA trifurcation segment. (**B**) Initial angiogram in working projection prior to the intervention. The posterior–anterior view is presented in the upper right corner. (**C**) After successful implantation of the p48MW HPC with the distal landing zone at the dominant superior trunk. The white arrow indicates the proximal landing zone, and the black arrow shows the distal ending of the FDS. Control injection confirmed the timely perfusion of the complete MCA territory without any delay in perfusion. (**D**) At the first angiographic follow-up, the opacification of the aneurysm remained unaltered (OKM A1); moreover, the side branches do not reveal any significant morphological changes. (**E**) At the second follow-up nine months after the FDS implantation, a significant reduction in aneurysm size with only residual opacification of the aneurysm neck was observed, corresponding to OKM C1. The perfusion of the covered MCA branches remained unaltered. (**F**) At the last available follow-up, 18 months after the treatment, the aneurysm was completely excluded from the intracranial circulation, while the covered M2 branches all remained intact.

Figure 2 exposes an exemplary case of aneurysm occlusion following implantation of the p48MW HPC flow diverter.

## 4. Discussion

FDS have become a well-established endovascular concept for cerebral aneurysms; however, there is only limited clinical experience with anti-thrombogenic coatings applied to the surface of FDS [8]. Furthermore, differences regarding treatment effects between bare metal (uncoated) and anti-thrombogenically coated FDS in small cerebral vessels have not been systematically investigated. Therefore, our study reviewed treatments with two frequently implanted, structurally comparable low-profile FDS, the SVB representing an uncoated FDS and the p48MW HPC as FDS with an anti-thrombogenic coating.

The architectures of the SVB and the p48MW HPC are similar; both are made of 48 nitinol strands with an inner platinum core. In contrast to the SVB, the p48MW is available with an anti-thrombogenic coating (hydrophilic polymer coating; HPC) that minimizes platelet adhesion together with clot formation in comparison to the uncoated version [22]. The hydrophilic coating was designed to imitate the glycocalyx of the intima of the vessel wall and to prevent inflammation [15]. In situations when dual anti-platelet therapy is not feasible, for example, after acute subarachnoid hemorrhage, the p48MW with HPC modification can be implanted under SAPT [17,23].

Although the structure of both stents is almost identical, the early hemodynamic effect on aneurysm perfusion and the time to definite occlusion differed considerably. More patients treated with the p48MW HPC showed reduced aneurysmal perfusion immediately after stenting (75.1%) compared to the SVB group (60%).

However, the follow-up results showed a diametrically opposed trend: only 27.3% of the aneurysms treated with the p48MW HPC were completely occluded at second follow-up compared to 64.4% completely occluded lesions in the SVB group. This suggests that further variables beyond the mechanical properties of the implants are at least equally important contributors to the therapeutic success.

The time to occlusion of an aneurysm after FDS treatment depends on a number of anatomical, hemodynamic and biological variables. In terms of angioarchitecture and hemodynamics, the magnitude of flow within the parent vessel, wall shear stress, dome to neck ratio, inflow angle, involvement of a bifurcation with or without competitive inflow (for example AcomA-complex vs. MCA-trifurcation) and the distance between aneurysm and parent vessel are known to influence the therapeutic effect after flow diversion [4,24,25,26,27,28]. Aside from that, the process of neointimalization, which biologically depends on disruption of endothelium at the landing zones, local inflammation, activation and binding of platelets to the implanted device together with the subsequent recruitment of circulating endothelial progenitor cells, dictates the timeframe of vascular healing after flow diverter treatment of an artery [29].

Anti-thrombogenic coatings and anti-platelet drugs both interfere with the cellular processes involved in the progress of vascular healing [30]. Since the anti-platelet medication was consistent in our patients, the hydrophilic coating of the p48MW constitutes the only elementary therapeutic difference between the two groups. As HPC inhibits platelet adhesion and activation, we consider the HPC surface modification of the p48MW to be a major factor involved in the longer time to aneurysm occlusion.

So far, meaningful evidence in this regard is scarce. Bhogal et al. demonstrated near-complete neoendothelialization 30 and 180 days after pCONus implantation, independent from the presence or absence of the HPC coating [31]. However, this study differs significantly from our analysis. First, the stent construct of the pCONus adjacent to the vessel wall is made of four wires only and therefore has a distinctly lower surface metal coverage than a FDS [32], requiring only a very small amount of neointima for coverage. Second, ASA and Clopidogrel were administered in Bhogal’s study, whereas the standard regimen at our neurovascular center comprised ASA and Ticagrelor.

Both Clopidogrel and Ticagrelor inhibit platelet aggregation via blockage of the P2Y12 receptor that is expressed on the cell surface. Apart from the anti-aggregant effect on platelets, only Ticagrelor is a strong inhibitor of the nucleotide receptor P2Y12, which mediates a variety of further, at this time not completely comprehended, mechanisms. Smooth muscle cells of the vessels and endothelial cells both carry P2Y12 nucleotide receptors. In vitro studies proved the inhibitory effect of P2Y12 nucleotide blockade on endothelial cell proliferation irrespective of the anti-aggregative effects [33]. Improved endothelial function was present after administration of Ticagrelor in patients suffering coronary arterial disease or chronic obstructive pulmonary disease [34]. This effect may be explained by a Ticagrelor-induced decrease in serum levels of the epidermal growth factor (EGF), the latter playing a major role in the genesis of endothelial dysfunction including atherosclerosis and vessel remodeling [35]. Apart from that, inhibition of the transmembrane protein equilibrative nucleoside transporter 1 (ENT1) mediated by ticagrelor has a major impact on the ticagrelor-driven anti-inflammatory effects [36].

As a consequence of the aforementioned, the choice of the second anti-aggregant certainly also influences the efficacy and speed of vascular healing after FDS implantation. However, as the majority of all patients in our study received Ticagrelor, the longer period until aneurysm occlusion in the p48MW HPC group is unlikely to be a consequence of a different DAPT medication scheme.

In our study, the p48MW HPC was preferentially implanted in the MCA (55.1%), whereas the SVB was mostly implanted into the ACA (56.9%). The hemodynamic situation in both territories varies significantly. For example, mean flows and mean velocities are significantly greater in the MCA than the ACA [37].

Furthermore, depending on the symmetry or asymmetry of the AcomA-complex, AcomA-aneurysms have potential inflow from two sources [38,39,40]. In case of proximal asymmetry, for example, a type 4 complex according to the classification of Krzyżewski and colleagues, where one dominant A1 segment predominantly supplies the ACA territory with only a minor contribution via the hypoplastic contralateral A1, to which it is connected to by an AcomA, the asymmetry actually facilitates aneurysm occlusion after unilateral hemodynamic intervention. More specifically, the flow diverter deployed in the dominant A1-A2 segment reduces ipsilateral inflow to the AcomA, and subsequently, the inflow via the contralateral, hypoplastic A1 becomes equally strong, resulting in a pressure equilibrium within the AcomA, and hence, aneurysm occlusion [4,41].

As a comparable hemodynamic situation with conflicting flows being present within the bifurcation itself does not occur under regular circumstances in the MCA bifurcation, but the M2 branches rather competitively drain blood from the MCA mainstem, so blood flow velocities usually remain stronger after FDS deployment. Therefore, a prolonged time to aneurysm occlusion after p48MW HPC may not only be the result of the anti-thrombotic coating, but also a consequence of the distinct, less favorable hemodynamic situation in the MCA compared to the ACA.

However, in our experience, a longer period between the implantation of a FDS and its complete coverage by neointima does not constitute a disadvantage, especially not when treating incidental aneurysms. When significant terminal branches arising in close proximity to the aneurysm are covered by the FDS for technical reasons, or when the hemodynamic situation in a bi- or trifurcation anatomy with only one inflow source is profoundly altered, the slower occlusion allows for more collateral vessels to develop, the prevention of ischemic events being supported by the DAPT.

In this regard, especially MCA-bifurcation aneurysms as a target for flow diversion are being discussed controversially with conflicting clinical evidence [42]. Cagnazzo et al. found 20% treatment-related complications with half of them having permanent clinical sequelae in an earlier study [43]. Diestro et al. reported 16% thromboembolic complications after FD of the proximal MCA bifurcation in a collective of patients who required retreatment after a failed first surgical or endovascular attempt [44]. Contrary to that, Salem et al. found that MCA-bifurcation aneurysms are treatable with much lower complication rates, the outcomes of their study being comparable to other, well consented FD locations [45]. Cimflova and coworkers contributed their experience with less than 9% significant complications after FD implantation in the MCA distal to the M1 segment [46].

### Limitations

The presented results were collected from a single center only, and consequently, are lacking a multicentric comparison and a greater study cohort. Furthermore, the difference regarding the predominant target vessels, namely the MCA for the p48MW HPC vs. the ACA territory for the SVB, represents a relevant bias in our study. Therefore, a prospective investigation with comparable treatment groups for each territory, even for the proximal ACA- and the proximal MCA-bifurcation, is wanted.

## 5. Conclusions

This study demonstrates a comparable safety profile of both FDS, the SVB and the p48MW HPC, for the endovascular therapy of peripheral intracranial aneurysms with differing early and late therapeutic outcomes when comparing both devices. While the SVB apparently achieved enhanced rates of early complete aneurysm occlusion, this may be influenced by its predominant use in the ACA territory in our study cohort. The rates of early aneurysm occlusion after treatment with the p48MW HPC were lower; however, the FDS was mostly deployed in the MCA territory, encountering different hemodynamic circumstances. With respect to the MCA perforators and the possible risk of thromboembolic events when covering side branches, the anti-thrombogenic coating of the p48MW HPC together with double anti-platelet therapy offers the advantage of prolonged occlusion times allowing for development of collateral vessels.

## Figures and Tables

**Table 1:** Study population.

	Total	SVB	p48MW HPC
**Number of patients**	*n* = 108	*n* = 60	*n* = 48
Gender			
Male	33	18	15
Female	75	42	33
Age in years	56 (18–84)	54.1 (18–83)	58.5 (31–84)
**Lesion characteristics**	*n* = 114	*n* = 65	*n* = 49
Measurements			
Neck width in mm		3.3	3.2
Dome width in mm		4.9	7.4
Dome height in mm		5.4	6.7
Morphology			
Saccular	110	65	45
Fusiform	1	0	1
Blister-like	2	0	2
Dissecting	1	0	1
Localization			
Internal Carotid Artery	21	14	7
Anterior Cerebral Artery	44	37	7
Middle Cerebral Artery	35	8	27
Vertebral Artery	5	4	1
Basilar Artery	8	1	7
Posterior Cerebral Artery	1	1	0
Treatment strategy	*n* = 108	*n* = 60	*n* = 48
Primary	62	30	32
Plug and Pipe	40	27	13
Revision	6	3	3
**Procedural aspects**			
Total number of implanted devices	128	72	56
Number of implanted SVB			
1	93	51	42
2	11	7	4
3 or more	9	2	2
Adjunctive devices	7	5	2
Anti-platelet regimen			
ASA 100 mg + Ticagrelor 180 mg daily	101	57	44
ASA 100 mg + Clopidogrel 75 mg daily	2	2	0
ASA 100 mg + Prasugrel 10 mg daily	2	1	1
ASA 200–400 mg daily	2	0	2
Prasugrel 10 mg daily	1	0	1
Additional oral anticoagulation	6	4	2

**Table 2:** Overview of the treated lesions.

	SVB	p48MW HPC
Total of treated lesions	*n* = 65	*n* = 49
A1-A2-Acom	29	6
Pericallosal Artery	8	1
M1 Segment	2	5
MCA Bifurcation/M2	6	22
Carotid T	1	1
ICA	9	5
Posterior Communicating Artery	4	1
V4 Segment/PICA Orifice	4	1
PCA/SCA incl. BA	2	7
Assessment of aneurysm occlusion		
Immediately after flow diversion	*n* = 65	*n* = 48 *
OKM A1	26	12
OKM A2-A3	31	33
OKM B	7	3
OKM C	1	0
OKM D	0	0
Last available angiographic follow-up	*n* = 59	*n* = 44
OKM A1	1	3
OKM A2-A3	3	5
OKM B	11	17
OKM C	5	5
OKM D	38	14
Technical adverse events	*n* = 6	*n* = 8
Retraction/Foreshortening/Dislocation	6	5
Insufficient opening	0	2
Secondary FDS kinking	0	1
Peri-interventional adverse events	*n* = 4	*n* = 1
Delay in distal perfusion	1	1
Thrombus formation/vessel occlusion	2	0
Extravasate	1	0
Clinical adverse events	*n* = 8	*n* = 8
Clinical manifest vasospasm/TIA	2	3
Symptomatic progredience in aneurysm size	1	0
Inflammation of aneurysm wall	0	1
Stent occlusion	2	1
Infarction	2	0
Intracerebral or subarachnoid hemorrhage	1	1
Other hemorrhage	0	1
Clinically manifest adverse events at last follow-up	3	2

* One case of a long-range dissecting aneurysm of the distal vertebral artery was not included for the assessment of the aneurysm occlusion rates using the O’Kelly–Marotta grading scale.

## Data Availability

Not applicable.
